# The impact of various surface treatments and Er-YAG laser irradiation on the bond strength of resin matrix CAD/CAM ceramics to resin cement

**DOI:** 10.1007/s10103-025-04460-5

**Published:** 2025-04-21

**Authors:** İlknur Usta Kutlu, Kaan Yerliyurt

**Affiliations:** Gaziosmanpaşa University, Tokat, Turkey

**Keywords:** Shear bond strength, Er-YAG laser, Resin matrix ceramic, Resin nanoceramic, Polymer infiltrated ceramic

## Abstract

**Purpose:**

The aim of the study was to assess the impact of various surface treatments, including an Er: YAG laser with different power outputs, on the shear bond strength (SBS) of different resin matrix CAD/CAM ceramic materials to resin luting cement.

**Materials and methods:**

Specimens from Lava Ultimate (LU) and Vita Enamic (VE) were categorized based on the surface treatment: no treatment, sandblasting (Sb), 10% hydrofluoric acid (Ac) etching, sandblasting followed by acid etching (Sb + Ac), and Er: YAG laser irradiation at 2 W and 3 W (L1, L2). Scanning electron microscopy (SEM) evaluation and roughness measurement of the samples were performed. Silane was applied to half of the specimens, resin cement was bonded and SBS testing was performed.

**Results:**

The SBS did not differ depending on the material (*P* = 0.081), but varied according to the surface treatment (*P* < 0.001). The highest mean SBS value was observed in the Sb + Ac (15.20) group. The highest roughness median was in Sb of VE, which was similar to Sb + Ac and Sb of all materials.

**Conclusion:**

Applying Sb and Ac together was more effective than applying them separately. Although 2 W and 3 W Er-YAG applications were ineffective for treating VE’s surface, 2 W Er-YAG applications can be suggested for LU.

**Clinical Relevance:**

The combined application of Sb and Ac is more effective than their individual applications and laser irradiation in terms of resin matrix CAD/CAM ceramics retention.

## Introduction

Nowadays, all ceramic CAD/CAM blocks are used more frequently in dentistry due to rising aesthetic demands. Material selection has become more significant as the diversity of CAD/CAM blocks has increased [1]. Polycrystalline ceramics and glass ceramics are the two subgroups of ceramic materials for CAD/CAM technology [2, 3]. Glass ceramics are materials that resemble composites, with the ceramic acting as reinforcing filler and the glassy phase serving as the matrix [4].

Although the glassy phase of glass ceramics increases their aesthetic properties, it also makes the structure brittle and tends to wear opposing teeth. To overcome the disadvantages of glass ceramics, resin-containing ceramic blocks have been introduced [5]. The two types of resin-containing ceramic CAD/CAM blocks can be distinguished as: resin-based ceramics (particle-filled composites), which have a polymer matrix including at least 80% nanosized ceramic filler particles (RNC), and hybrid ceramics, which are composed of a ceramic network infiltrated with a polymer (PIC) [6]. In order to form the PIC, a polymer takes the place of the fragile glass phase. PIC networks are made up of two continuous interpenetrating networks as opposed to typical composites, which are made up of one continuous phase filled with inorganic particles. There are two types of networks: one made of ceramic material and the other of polymer [7]. With their dual-network structure, both of these materials combine the advantageous properties of ceramic and composite materials.

Compared to glass ceramics and conventional ceramics, resin-containing ceramic materials offer several advantages, such as easier milling and adjustment, a more precise imitation of dentin’s elasticity, better polishability, intraoral reparability, and the lack of a firing requirement [7–11]. Additionally, compared to direct composite restorations, indirect composite restorations have some advantages, such as decreased polymerization stress and residual monomer, enhanced physical characteristics, enhanced proximal surface contouring, enhanced resistance to wear and coloring, and biocompatibility [12, 13]. Vita Enamic (VE) is the commonly used PIC material, whereas Lava Ultimate (LU) is the commonly used resin nanoceramic [1, 12, 14, 15]. Although the crown indication of LU was removed by the manufacturer on June 12, 2015, inlays, onlays, and veneers can be constructed as indicated [16].

A crucial stage as important as material selection that impacts treatment effectiveness and lifetime is the adherence of indirect restorations to the tooth structure and resin cement [17]. Inadequate bonding can result in poor marginal fit, micro-leakage, low retention, and low fracture strength [9, 10]. Along with micromechanical interlocking, chemical bonding to the restoration surface is essential for a stable bond [3, 18]. Silanization can develop a chemical bond between the hydrophobic resin cement and the hydrophilic ceramic surface [2, 19].

Cements, silane coupling agents, and priming agents are also infused into microretentive bonding surfaces to produce micromechanical interlocking [20]. In order to increase microretentive areas and enhance hydrophilicity, various surface treatments, such as acid application [1, 3], silica coating [14, 21, 22], mechanical abrasion with diamond rotary instruments [12, 23], sandblasting [1, 3, 9, 14], laser applications [3, 9, 10, 21–24], non-thermal atmospheric plasma application [25], and combinations of any of these methods, are applied to the restoration [25].

For ceramic etching, the use of an acid-based gel has been demonstrated to offer improved retention and is advantageous for chairside application [26]. Through the formation of pits on the material during the selective elimination of the glassy phase from the ceramic matrix, hydrofluoric acid (HF) serves to promote micromechanical bonding [23]. However, the lack of a glassy phase in indirect resin composite blocks may prevent HF acid etching [5]. Also, due to its volatility and toxicity, HF acid poses a risk to human health [22].

Sandblasting can produce a clean, rough surface for adhesion [27, 28]. It is anticipated that sandblasting surface preparation varies based on a wide variety of settings, including particle size, pressure, distance from the surface, working duration, and impact angle [29]. Applying excessive pressure could cause the material’s surface and subsurface to crack, weakening the material’s cohesive strength at the surface and ultimately leading to failure at the interface [5].

Erbium-doped yttrium aluminum garnet (Er: YAG) laser [5, 30], erbium, chromium-doped yttrium, scandium, gallium, and garnet (ErCr: YSGG or ECY) laser [1, 3, 9], neodymium-doped yttrium aluminum garnet (Nd: YAG) laser [21, 22], and femtosecond lasers [31] are laser types used for surface treatment of CAD/CAM ceramic blocks in dentistry. Er: YAG is one of the most promising laser types since its wavelength coincides with the water’s absorption peak [29].

During laser treatment, internal tensions can cause damage to the materials due to rapid local temperature changes that occur throughout the heating and cooling stages. Therefore, utilizing the proper laser operating parameters, especially power output, is essential [5, 26]. Although thermomechanical effects of the Er: YAG laser have been observed on substrates, there is still uncertainty over the potential advantages of this laser type.

In this study, it is aimed to evaluate the effect of Er: YAG laser pretreatment and conventional treatments of acid etching, sandblasting, and silanization in comparison to the control group on the shear bond strength of resin matrix ceramic CAD/CAM blocks.

The null hypothesis was that;

1. There would be no statistically significant difference in the bond strength of the tested resin matrix ceramic blocks.
2. Different surface treatments and silanization would not affect the bond strength values.

## Materials and methods

**Fig. 1:**
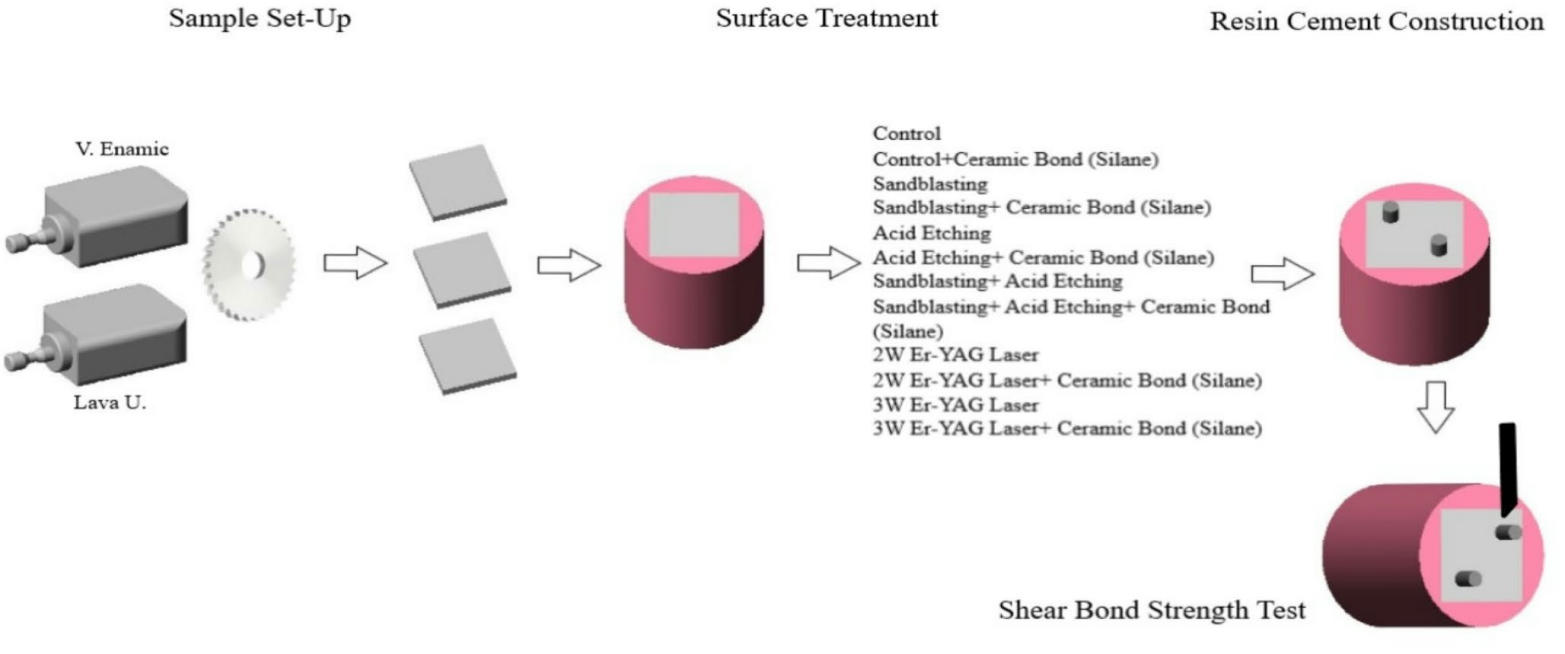
Schematic illustration of the study setup

In this in-vitro study, two distinct resin matrix ceramic (RMC) blocks, resin nanoceramic Lava Ultimate (LU) and polymer infiltrated ceramic network Vita Enamic (VE), were employed. Table 1 illustrates the brands, manufacturers, and chemical compositions of the utilized materials. Additionally, a schematic representation of the study setup is provided in Fig. 1.

**Table 1:** Materials used in the study

Material	Type of RMC	Composition	Manufacturer
Lava Ultimate	RNC; Resin Nano Ceramic	Organic part: Bis-GMA, Bis-EMA, UDMA, TEGDMA Inorganic part: 80 wt% silica and zirconia nanoparticles and zirconia/silica nanoclusters	3 M ESPE, St. Paul, MN, USA
VITA Enamic	PICN; Polymer-Infiltrated Ceramic Network	Organic part: UDMA, TEGDMA Inorganic part: 86 wt% glass ceramic (SiO_2_, Al_2_O_3_, Na_2_O, K_2_O, and other oxides)	VITA Zahnfabrik, Bad Säckingen, Germany
Ceramic Bond	Coupling Agent (silane)	Organic acid, methacrylated phosphoric acid ester, 3-methacryloxypropyltrimethoxysilane and acetone	Voco GmbH, Cuxhaven, Germany
Maxcem Elite	self-adhesive dual curing resin cement	HEMA, MEHQ, CHPO, TiO_2_, Uncured Methacrylate Ester Monomers, pigments. pH approximately 2.5	Kerr Corp., Orange, CA, USA
Angelus	Acid etchent	HF acid 10%	Angelus, Londrina, Brezilya

RMC: Resin matrix ceramic, Bis-GMA: bisphenol A glycol dimethacrylate, UDMA: urethane dimethacrylate, Bis-EMA: bisphenol A ethoxylated dimethacrylate, TEGDMA: triethyleneglycol dimethacrylate, HEMA: 2 hydroxyethyl methacrylate, MEHQ: 4-methoxyphenol, CHPO: cumolhydroperoxid

CAD-CAM blocks were cut into 2 mm thick rectangular sections on a water-cooled cutting machine (Micracut 200, Metkon, Turkey), and 48 specimens for SBS testing and 6 for SEM were obtained from each material (a total of 108). Subsequently, they were embedded in a self-cured acrylic resin (Integra; BG Dental, Ankara, Turkey) with the help of a cylindrical teflon pipe, ensuring that the surfaces intended for testing were oriented upwards. Each specimen underwent polishing with consistent water irrigation using wet silicon carbide paper (Metkon Instruments Inc., Bursa, Turkey) of 600, 800, and 1000 grit, respectively. Specimens of each material were randomly allocated into six groups based on the applied surface treatment method (*n* = 8).

Group 1 (C): The control group does not receive any surface treatment.

Group 2 (Sb): The surfaces underwent airborne-particle abrasion using 50 μm aluminum-oxide (Al_2_O_3_) particles (Renfert GmbH, Germany) from a distance of 10 mm for 20 s, with a pressure of 2 bar (Basic Eco, Renfert GmbH, Germany). The specimens were rinsed in distilled water for 20 s and air-dried.

Group 3 (Ac): The specimens underwent etching with 10% hydrofluoric acid (Angelus, Londrina, Brazil) for 60 s, followed by thorough rinsing with water spray for 20 s.

Group 4 (Sb + Ac): Following the same surface abrasion procedure as conducted in Group 2, HF acid was then applied as outlined in Group 3.

Group 5 (L1): The specimens were treated using an Er: YAG laser (KaVo K-E-Y Lazer 3+, KaVo, Germany) in short pulse mode, with a wavelength of 2940 nm, an output power of 2 W, a pulse energy of 200 mJ, and a pulse frequency of 10 Hz. The laser was applied in a sweeping motion, maintaining a distance of 8 mm above the surface, for a total of 20 s using the laser contra-angle handpiece 2060, with 60% air and 50% water flow.

Group 6 (L2): The specimens were treated using an Er: YAG laser (KaVo K-E-Y Lazer 3+, KaVo, Germany) in short pulse mode, with a wavelength of 2940 nm, an output power of 3 W, a pulse energy of 300 mJ, and a pulse frequency of 10 Hz. The laser was applied in a sweeping motion, maintaining a distance of 8 mm above the surface, for a total of 20 s using the laser contra-angle handpiece 2060, with 60% air and 50% water flow.

All specimens were subjected to ultrasonic cleaning (Pro-Sonic 600; Sultan Healthcare, USA) for 5 min to eliminate impurities and air-dried. Subsequently, one specimen from each group was examined using a scanning electron microscope (SEM) at ×1000 magnification (Hitachi SU1510, Tokyo, Japan). Surface roughness measurements (*n* = 8) were conducted using a contact profilometer (Surtronic 25, Taylor Hobson Ltd., Leicester, England). The profilometer was calibrated before measurements were taken. In various directions, five measurements were made (evaluation length: 4 mm, Gaussian filter cutoff: 0.80 mm). The treated surfaces’ average roughness values (Ra) were measured in micrometers (µm) [32].

Each group was further divided into two subgroups, wherein silane (Ceramic Bond, Voco, Germany) was applied to half of the specimens for 60 s. Following silane application, specimens were dried using oil-free, gentle airflow.

Cylindrical resin cement (Maxcem Elite Kerr Corp., USA) with a diameter of 2.8 mm and a height of 3 mm was built up with the help of two cylindrical polyethylene tubes on each specimen surface. After curing with a light-emitting diode (LED) curing device (Valo, Ultradent) at 1200 mW/cm^2^ for 40s, the polyethylene tube was cut out with a scalpel. Prior to conducting the SBS test, the bonded specimens were stored in distilled water at 37 °C for 24 h.

A universal test apparatus (Shimadzu, MWG-5 kNA series; Shimadzu Corp.) was used to perform the SBS test. Axial force application was carried out between the specimens and the resin interface using knife-edge-shaped equipment at a crosshead speed of 1 mm/min until failure occurred. The SBS in megapascal was determined by dividing the force at failure, measured in Newtons, by the surface area (mm^2^) using the following formula: SBS (MPa) = Force (N) / Area (mm^2^)The results were evaluated statistically.

A single operator examined the specimens utilizing a stereomicroscope (Carl Zeiss, Göttingen, Germany) at 40X magnification to determine the mode of failure. The failure modes were categorized as follows: Adhesive (no resin cement on the surface of the CAD/CAM block), cohesive (the entire fracture surface is nearly entirely covered with resin cement), and mixed (some adhesive cement on the CAD/CAM block).

### Statistical analysis

Data were analyzed with the IBM SPSS V23 and JAMOVI V2.3.21 0.21 programs. Compliance with normal distribution was examined according to Shapiro-Wilk and kurtosis-skewness values. A three-way ANOVA was used to compare normally distributed bond strength values according to material, surface treatment, and silane application, and multiple comparisons were examined with the Tukey’s Honestly Significant Difference (HSD) test.

Comparison of non-normally distributed roughness values according to material and surface treatment was examined with two-way Robust ANOVA using the Walrus package, and multiple comparisons were examined with Bonferroni correction. The significance level was set at *P* < 0.050.

## Results

The mean shear bond strength did not differ depending on the material, regardless of surface treatment or silane application (*P* = 0.081) (Table 2). VE had a mean SBS of 11.21, while LU had a similar mean SBS of 10.36. Irrespective of material type and silane application, the mean SBS varied according to the main effect of surface treatment (*P* < 0.001). The highest mean SBS value was observed in the Sb + Ac (15.20) group, followed by Sb (11.89), Ac (11.59), L1 (9.86), and L2 (8.72), respectively (Table 3). When the partial eta square values were examined, the surface treatment had the highest effect on bond strength (ηp2 = 0.3899), while the main effect of the material had the lowest effect (ηp2 = 0.018) (Table 2).

**Table 2:** Comparison of bond strength

	F	*P*	ηp 2
Material	3.080	0.081	0.018
Surface Treatment	21.430	**< 0.001**	0.389
Silane	5.470	**0.021**	0.032
Material * Surface Treatment	2.520	**0.031**	0.070
Material *Silane	7.180	**0.008**	0.041
Surface Treatment * Silane	2.770	**0.020**	0.076
Material * Surface Treatment * Silane	0.790	0.561	0.023

F: Analysis of variance, R^2^=%47,71, Adjusted R^2^=%40,55, ηp 2: Partial eta squared

**Table 3:** Descriptive statistics and multiple comparison results of shear bond strength (Mpa)

Surface Treatment	Silane	Material		Mean
		VE	LU	
C	Silane (-)	7.66 ± 3.03	5.84 ± 1.10	6.75 ± 2.39^E^
	Silane (+)	10.28 ± 5.32	6.10 ± 0.63	8.19 ± 4.25^DE^
	Mean	8.97 ± 4.40^CDE^	5.97 ± 0.88^E^	7.47 ± 3.47^c^
Sb	Silane (-)	13.71 ± 4.62	13.85 ± 3.20	13.78 ± 3.84^AB^
	Silane (+)	11.95 ± 4.58	8.05 ± 0.95	10.00 ± 3.78^BCDE^
	Mean	12.83 ± 4.53^ABC^	10.95 ± 3.77^BCD^	11.89 ± 4.21^b^
Ac	Silane (-)	11.45 ± 4.67	12.73 ± 2.58	12.09 ± 3.70^BCD^
	Silane (+)	11.10 ± 2.97	11.05 ± 3.73	11.08 ± 3.26^BCD^
	Mean	11.28 ± 3.79^BCD^	11.89 ± 3.22^BCD^	11.59 ± 3.47^b^
Sb + Ac	Silane (-)	17.01 ± 5.30	16.44 ± 3.80	16.73 ± 4.46^A^
	Silane (+)	15.28 ± 2.58	12.08 ± 3.37	13.68 ± 3.34^ABC^
	Mean	16.14 ± 4.12^A^	14.26 ± 4.14^AB^	15.20 ± 4.17^a^
L 1	Silane (-)	7.30 ± 2.25	12.21 ± 3.54	9.76 ± 3.83^CDE^
	Silane (+)	10.30 ± 3.79	9.61 ± 2.47	9.95 ± 3.11^BCDE^
	Mean	8.80 ± 3.38^DE^	10.91 ± 3.24^BCD^	9.86 ± 3.43^bc^
L 2	Silane (-)	9.64 ± 2.25	8.37 ± 2.83	9.00 ± 2.55^DE^
	Silane (+)	8.85 ± 3.32	8.02 ± 1.51	8.43 ± 2.53^DE^
	Mean	9.24 ± 2.77^CDE^	8.20 ± 2.20^DE^	8.72 ± 2.52^c^
Mean	Silane (-)	11.13 ± 5.04^A^	11.57 ± 4.52^A^	11.35 ± 4.77
	Silane (+)	11.29 ± 4.19^A^	9.15 ± 3.05^B^	10.22 ± 3.80
	Mean	11.21 ± 4.61	10.36 ± 4.03	10.79 ± 4.34

VE: Vita Enamic, LU: Lava Ultimate, C: Control, Sb: Sandblasting, Ac: Acid Etching, Sb + Ac: Sandblasting + Acid etching, L1: 2 W laser irridation, L2: 3 W laser irridation

a-c: There is no difference between surface treatment main effects with the same letter, A-E: There is no difference between interaction groups with the same letter, mean ± Standart Deviations

The mean bond strength varied depending on the interaction of material and surface treatment (*P* = 0.031). As the multiple comparison results of material and surface treatment interaction groups are presented in Table 3, the highest mean SBS value was 16.14 in the Sb + Ac group of VE, which was similar to the Sb group of VE. The Ac, Sb, and control groups were also similar in VE. The highest mean value of LU was 14.26 in the Sb + Ac group, which was similar to the Sb, Ac, and L1 groups of LU. The SBS of control groups and L1 or L2 treated all VE and LU groups were similar, except for L1 in the LU group. L1 in the LU group was statistically greater than the control group. The Sb and Ac groups were statistically similar in both materials, which were similar to the control group in VE and higher than the control group in LU.

Regardless of the surface treatment, the mean SBS of the silane applied VE groups (11.29) was statistically consistent with the VE groups without silane application (11.23). The mean SBS of the silane-applied LU groups (9.15) was statistically different (*P* = 0.008) and lower than the LU groups to which silane was not applied (11.57). According to the main effect of silane, it could be stated that silane application lowers the bond strength (*P* = 0.021). The mean value of the two materials without silane application was 11.35, and the mean value of the silane applied groups was 10.22. The mean SBS did not differ according to material x surface treatment x silane interaction (*P* = 0.561).

### Sufface roughness results

Roughness medians differed according to material type, regardless of surface treatment (*P* = 0.047) (Table 4). The median of VE was (0.710) higher than the median of LU (0.470) (Table 5). Roughness medians also differed according to the main effect of surface treatment (*P* < 0.001). In all surface treatment types, the median roughness was higher than the control group, regardless of material type. The groups with the greatest roughness medians, Sb and Sb + Ac (2.010 and 1.885, respectively), were similar to each other.

**Table 4:** Comparison of surface roughness

	F	*P*
Material	3.94	**0.047**
Surface Treatment	131.17	**< 0.001**
Material * Surface Treatment	55.86	**< 0.001**

*Robust ANOVA, median method was used as comparison

**Table 5:** Descriptive statistics and multiple comparison results of roughness values (πm)

Surface Treatment	Material		Mean
	VE	LU	
C	0.230 (0.200–0.270)^A^	0.225 (0.160–0.300)^A^	0.230 (0.160–0.300)^b^
Sb	2.080 (1.750–2.340)^B^	1.880 (1.640–2.270)^BD^	2.010 (1.640–2.340)^a^
Ac	0.690 (0.540–0.740)^C^	0.180 (0.160–0.470)^A^	0.505 (0.160–0.740)^c^
Sb + Ac	1.800 (1.680–2.070)^D^	1.980 (1.590–2.490)^BD^	1.885 (1.590–2.490)^a^
L 1	0.305 (0.220–0.460)^E^	0.430 (0.230–0.680)^F^	0.355 (0.220–0.680)^d^
L 2	0.855 (0.550–1.660)^C^	0.540 (0.310–0.730)^F^	0.610 (0.310–1.660)^e^
Mean	0.710 (0.200–2.340)	0.470 (0.160–2.490)	0.565 (0.160–2.490)

VE: Vita Enamic, LU: Lava Ultimate, C: Control, Sb: Sandblasting, Ac: Acid Etching, Sb + Ac: Sandblasting + Acid etching, L1: 2 W laser irridation, L2: 3 W laser irridation

Median roughness varied depending on the interaction of material and surface treatment (*P* < 0.001) (Table 4). The highest median value, 2.080, was obtained from the Sb group of VE, which was similar to the Sb and Sb + Ac groups of LU (Table 5). The L1 and L2 groups of both LU and VE had lower median roughness than all groups of Sb and Sb + Ac. While the L1 and L2 groups of LU were similar to each other, L2 of VE was higher than L1 of VE. The lowest median value, 0.180, was obtained from the Ac of LU, which was the only group statistically similar to the control group (Table 5).

### Sem results

**Fig. 2:**
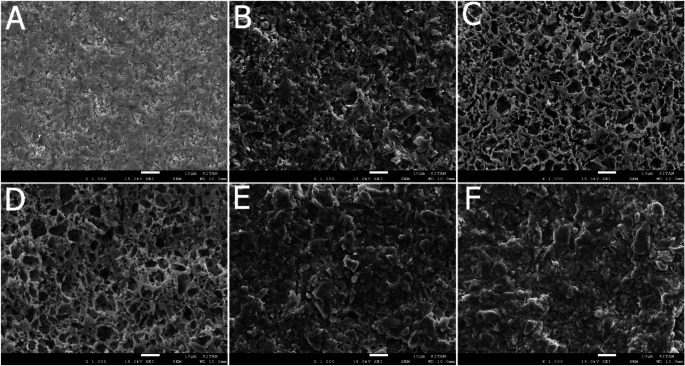
SEM images of the Enamic groups at x 1000 magnification

**Fig. 3:**
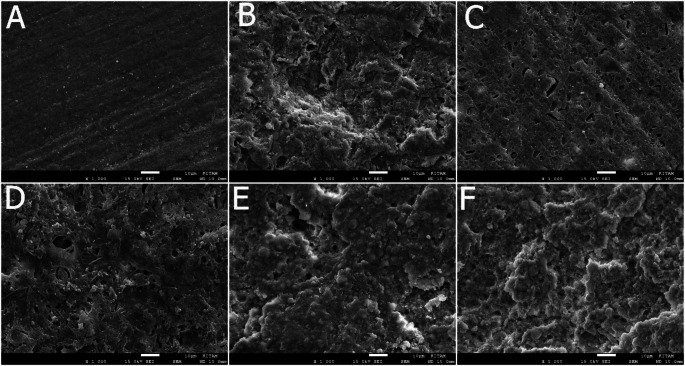
SEM images of the Lava groups at x 1000 magnification

Significant alterations in surface morphology were observed across all groups in VE compared to the control group. Particularly, a honeycomb appearance was noted in the Ac and Sb + Ac groups of VE (Fig. 2). L1, L2, and Sb groups exhibited similar characteristics. In contrast, the change in the Ac group of LU was less pronounced compared to VE, showing only minimal differences from the control group (Fig. 3). Similarly, L1, L2, and Sb groups in LU displayed similar features.

**Fig. 4:**
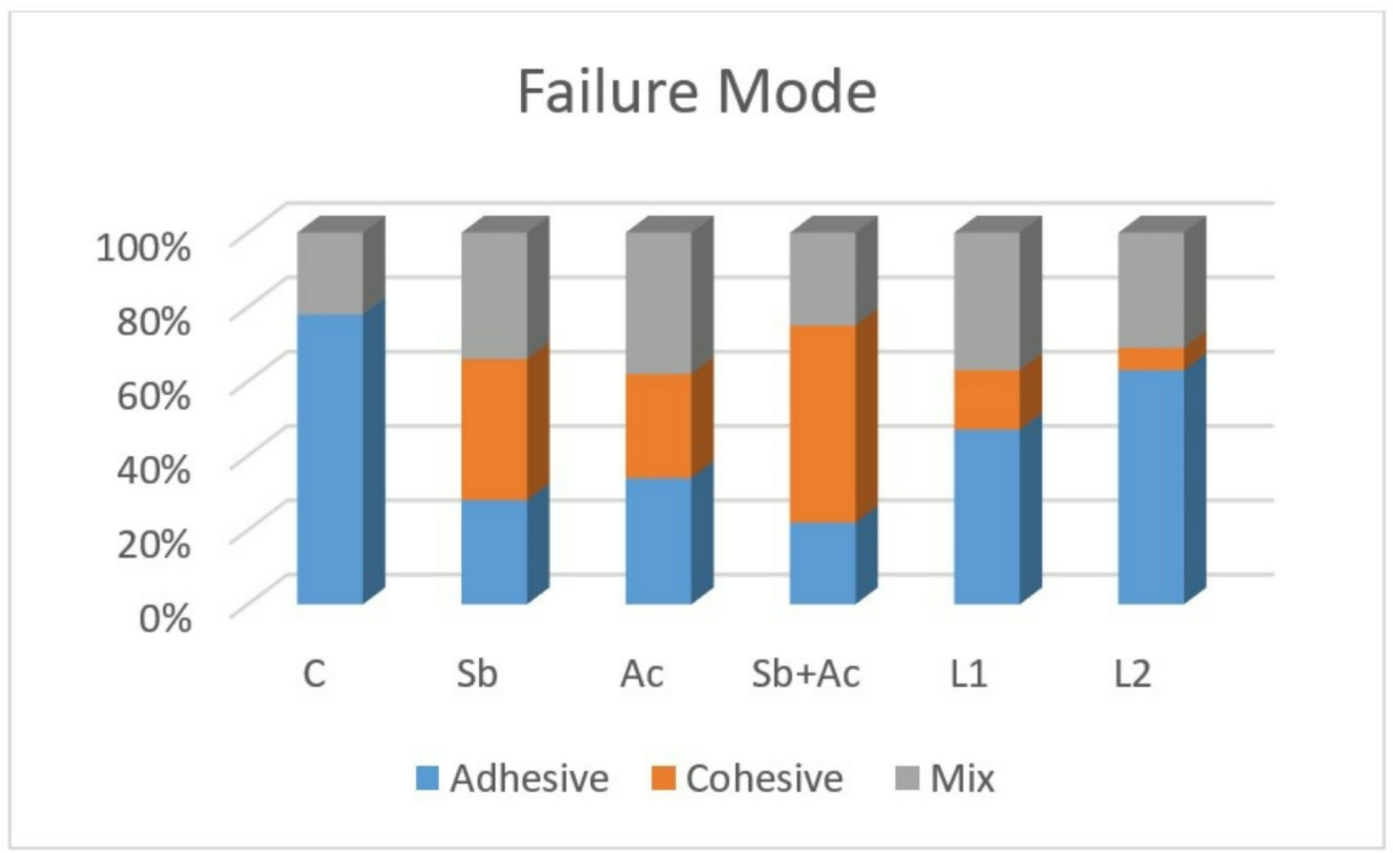
Failure type results following SBS test

According to the fracture mode analysis, adhesive failure was mostly seen in all groups; however, it was most noticeable in the C, L1, and L2 groups. The percentage distribution of failure modes for the tested materials is depicted in Fig. 4. Cohesive failures were exclusively observed within the resin cement, with a higher occurrence noted in the Sb + Ac groups compared to other groups.

## Discussion

This study investigated the effects of different surface treatment methods and silanization on the shear bond strength of self-adhesive resin cement to resin matrix ceramic CAD/CAM blocks. According to the findings of the study, the first null hypothesis was accepted, as there was no difference between VE and LU. Although 2 W and 3 W Er-Yag laser applications in two materials were found to be no different from the control group, except for L1 in the Lava groups. Sandblasting followed by the HF acid etching group (Sb + Ac) showed the higher SBS values than the control group in both materials, so the second null hypothesis was rejected.

Although resin-based ceramic blocks with organic and inorganic matrixes differ from ceramic materials, which contain only mineral or inorganic compounds, they can be classified as ceramics due to their inorganic content of over 80% in accordance with the American Dental Association (ADA) Code on Dental Procedure and Nomenclature in 2013 [25, 33].

The total weight% of nanoceramic material in LAVA Ultimate is about 80%, made up of nanomer and nanocluster fillers [1]. A network of 86% feldspar ceramic material is infiltrated with 14% resin polymers to generate Vita Enamic, an interpenetrating-phase composite material [34]. There was no statistical difference in the SBS values of VE and LU, despite the varied content of these two materials, in line with the study by Çelik et al. [22].

The high heat and pressure used in the production of CAD/CAM blocks and a higher degree of conversion result in a surface devoid of carbon-carbon double bonds [27]. In order to improve bonding strength, surface modifications can be required. HF acid is considered the most effective surface pretreatment method for ceramic indirect restorative materials [18, 35]. Kilinc et al. [11] concluded that HF etching is more effective for ceramic/glass ceramics than resin ceramics in achieving a durable bond. Moreover, it is still the gold standard for glass ceramic surface treatment [36]. When HF acid is applied to a glass ceramic surface, it reacts with silicone dioxide, removing the vitreous matrix and revealing the crystalline structure. This surface roughness reduces the contact angle, which enhances ceramic wettability and increases the surface area for bonding agents [3, 17]. Indirect resin composite blocks, however, lack a glassy phase. Queiro et al. [37] stated that 90 s 5% HF acid etching is more effective in roughening VE, while 20–40 s 10% HF acid etching is more effective for LU. That can be attributed to the glassy phase of VE, which is more than LU.

Sandblasting improves the mechanical bonding by exposing the filler particles and increasing the surface area [1, 5]. According to the results of a systematic review and meta-analysis conducted by Moura et al. [15], the majority of research found that HF is the optimal surface treatment for VE, and airborne particle abrasion with Al_2_O_3_ for Lava Ultimate in terms of bond strength. According to Şişmanoğlu et al. [34], higher bond strength values are caused by airborne particle abrasion as the composite component increases, while higher bond strength values are produced by HF treatment when the material composition contains more ceramic.

In the current study, the best surface treatment for both materials was Sb + Ac, where sandblasting and acid application were combined. In line with the study of Sagsoz et al. [38], the Sb and Ac groups of VE were similar to the control group. In contrast, Sb and Ac were similar and more than control in LU. While investigations [28] assessing both HF acid etching and sandblasting have demonstrated enhancements in the bond strengths of VE and LU, Turker et al. [36] evaluated the bond strengths of VE and LU to resin cement and found that there was no statistically significant difference between the sandblasting and control groups. Tekçe et al. [39] applied sandblasting to LU and VE for varying durations, revealing that LU exhibited similarity to the control group across all durations and concluded that, as sandblasting time increases, roughness increases, but bond strength decreases over 30 s. Also, the particle size of the sand, pressure and the distance of the probe may affect the bond strength [29, 36]. Sandblasting may result in material loss or excessive cavities on the ceramic surface, consequently reducing its flexural strength [5, 10, 21, 22]. Similarly, HF etching has been shown to diminish the flexural strength of lithium disilicate ceramics, depending on the duration and concentration of the etching applied [35].

Some of the studies suggest silanization following HF etching or sandblasting in resin matrix ceramics [28, 34]. In contrast, several researchers have concluded that the application of silane does not result in a notable increase in bond strength [10, 40]. Silane is a common coupling agent that promotes chemical bonding and interacts with both the organic phase of the composite resin and the crystalline phase of the ceramic [23]. In addition to improving the formation of siloxane bonds, silane coupling agents with a coupler and a weak acid also make the ceramic surface more wettable [18]. Silane molecules undergo a reaction with water, leading to the formation of three silanol groups from the methoxy groups. Then, the silanol groups react with the silica surface to generate a siloxane network. Through a process known as free radical polymerization, the monomeric ends of the silane molecules interact with the methacrylate groups of the resins [17].

The LU (20%) has a higher amount of resin than VE (14%). Triethylene glycol dimethacrylate (TEGDMA), which is more prevalent in VE compared to LU, demonstrates higher conversion rates and lower levels of residual monomer, thereby posing challenges for bonding [23]. In the current study, silane application exhibited no variation in the bond strength of VE but decreased the bond strength of LU. Consistent with our findings, Günal et al. [9] reported that silane diminishes the bond strength of all resin matrix ceramics, such as Lava, with the exception of Vita Enamic. Bahadır et al. also reported that silane was ineffective in LU but noted an increase in bond strength in certain VE groups, attributing this discrepancy to variations in silica content and microstructure between the two materials.

In the conclusion of the study by Kılıç et al. [11] laser iridiation was suggested as an alternative surface treatment method to HF etching and sandblasting. During laser application, the water supplied by the laser system absorbs the laser beam, leading to micro-explosions that occur in less than a picosecond and result in irregular changes on the laser-irradiated surface [5, 23], This process removes inorganic content, thereby enhancing the micromechanical interaction between the resin cement and ceramic surface [3, 5]. The laser parameters, including energy, power outputs, repetition rate, pulse length, and application time, can also influence surface alteration through material ablation [24].

A systematic review and meta-analysis on the bond strength of ceramic materials demonstrated that all laser types yield higher bond strengths in comparison to non-treated surfaces. However, there were no significant differences when compared to sandblasting [24]. In contrast, Özarslan et al. [41], found that the SBS of the VE and LU brackets was greater in the groups treated with HF and sandblasting compared to laser application. Shiu et al. [42] observed that Er: YAG laser treatment resulted in poor bond strength regarding the SBS of ceramic to resin cement. Turker et al. [36] evaluated the SBS of VE and LU with resin cement and did not observe any difference among the Al2O3 sandblasting, laser, and control groups. Similarly, in the present study, there was no difference between the 2 W Er-Yag (L1) and 3 W Er-Yag (L2) irradiated VE groups and the control group. This may be due to the fact that both power outputs are not strong enough to provide sufficient microdepths of the irregularities for VE, as it has greater microhardness than LU [9]. In the laser treatment groups, only L1 of LU showed significantly greater SBS than the control group, and L2 of LU was similar to the control group.

Explosive vaporization occurs during composite resin ablation at temperatures exceeding 300 °C [5]. High power settings were associated with inferior bond strength values by Gökçe et al. [26], who also noted a poorly attached heat-damaged layer on the inner surface, contrasting with the strong bond to the resin cement on the outer surface. The bonding behavior of VE (2 W, 3 W) and LU (3 W) may be attributed to the inadequate attachment of this layer to the underlying layers.

Motevasselian et al. [29] applied 2 W and 3 W Er-Yag to the VE, examined microtensile bond strength, and found it similar to the control group. They suggested two possible reasons for this: either the low energy density of the irradiating laser or the damaged superficial layer of ceramic separated from the underlying ceramic during the bond strength test. Low SBS values of VE, which irridiated with 3 W and 6 W power output Er-Yag lasers, were also attributed to the fragmentation by El-Damanhoury et al. [5] In line with these studies, Garshasbzadeh et al. [30] utilized Er-yag lasers with varying output powers on indirect composites, conducted SEM evaluations, and noted that those with 2 W, 3 W, and 4 W output power resulted in increased roughness, whereas those with 5 W, 6 W, and 7 W output power exhibited reduced roughness. Based on the roughness measurements from the present study, there was no discrepancy between the 2 W and 3 W power outputs in LU. However, in VE, the 3 W laser application generated greater roughness compared to the 2 W. Nonetheless, when examining the SBS values, no distinction was evident between L1 and L2 in the VE group. Although the median roughness values of Sb and Sb + Ac in the current study exhibit the highest values parallel to the SBS values, as stated by numerous studies [10, 14, 28, 31], roughness does not guarantee enhanced bonding strength. Similarly, in the SEM analysis of the study, the L1 and L2 groups of both materials exhibited a rougher surface appearance compared to the control group, which was inconsistent with the SBS values. Inadequate microdepths of the porosities created by laser power settings, which led to poor SBS, may be the cause of these outcomes.

Widespread used self-adhesive cements bond to dentin and veneer, minimize postoperative sensitivity, and reduce the potential risk of complications with reduced application steps [2]. With indirect restorations, dual-curing self-adhesive resin cement was found to have a stronger bond than self-curing self-adhesive resin cement [28, 43]. Therefore, dual-curing self-adhesive resin cement was preferred to use in the study. The coupling agent utilized in the investigation, Ceramic Bond, is a silane (3-methacryloxypropyltrimethoxysilane). containing adhesive. Şişmanoğlu et al. [34] and Awad et al. [17] asserted that silane included in universal adhesives does not serve as a substitute for additional silane application. Subsequent research could explore comparing the impacts of silane-containing adhesives with separate applications of silane and adhesive.

Although the SBS test is frequently employed for in vitro studies of resin ceramics, there are several restrictions regarding the material’s inhomogeneous stress distribution that cause cohesive failure and lead to faulty interpretations [31]. In the present study, cohesive failures within resin cement were observed in the sandblasting followed by acid etching groups (Sb + Ac), whereas mainly adhesive problems were noted, particularly in the control and laser groups, at the interface between the ceramic and resin cement.

Due to the water absorption, which leads to the hydrolytic breakdown of methacrylate monomers, the adhesion and mechanical properties of resin matrix blocks may change more quickly than those of ceramics over time [25]. Since the restorations function in a humid and thermally variable oral environment, not taking the hydrolysis of the bonding area into consideration and not using the thermal cycle can be a limitation of the study.

## Conclusions

The following findings could be made within the limitations of this in vitro investigation:

1- When applied individually, both Ac and Sb yielded similar SBS results for both materials. However, the combined application of Sb and Ac proved to be more effective than their individual applications in terms of both bonding strength and surface roughness.

2- The use of 2 W Er-Yag for laser treatment may be advised for LU, even though the laser application demonstrated a consistent main effect across both 2 W and 3 W power outputs, with mean SBS values similar to the control group.

3- Silane containing Ceramic Bond did not affect SBS of VE and adversely affected LU.

## Data Availability

The data sets used and/or analysed during the current study are available from the corresponding author upon reasonable request.
